# Letter to the Editor: ‘Diabetes and technology – an update for the general physician’

**DOI:** 10.1016/j.clinme.2026.100578

**Published:** 2026-03-27

**Authors:** Prajakta Udmale, Vaishali Khobragade, Aparna Dixit

**Affiliations:** Department of Human Anatomy, Dr. DY Patil Homoeopathic Medical College and Research Centre, Dr. DY Patil Vidyapeeth (Deemed to be University), Pimpri, Pune, India

Dear Editor,

Khin *et al*’s new article ‘Diabetes and technology – an update for the general physician’ piqued our interest, as it gives a contemporary and clinically relevant overview of evolving diabetes technology and its application in general medical practice. The authors deserve credit for providing a succinct but comprehensive overview of continuous glucose monitoring (CGM), continuous subcutaneous insulin infusion (CSII) and hybrid closed-loop (HCL) devices, as well as practical advice for inpatient and postoperative care. The emphasis on real-world obstacles experienced by non-specialist physicians, such as a lack of familiarity, low confidence and the need for structured instruction, is particularly significant and resonates with global concerns about technology adoption in primary care.

The article’s strengths stem from its pragmatic approach, which includes comprehensive descriptions of device functionality, clinical indications and troubleshooting procedures in acute settings. The addition of scenarios requiring capillary blood glucose validation, as well as guidance on perioperative and radiographic care, improves its usefulness in hospital settings. Furthermore, studies suggesting benefits in HbA1c, time-in-range, and reduction in hypoglycaemia using automated insulin delivery systems lend support to the topic of HCL systems’ expanding role and impact on glycaemic outcomes.^1^, ^2^ Furthermore, the clinical value of CGM parameters like time-in-range has been extensively established and correctly highlighted in the context of current diabetes care.^1^

However, certain limits should be considered. First, while the assessment effectively targets high-income healthcare systems such as the NHS, it has limited global relevance, particularly in low- and middle-income nations where access, pricing and infrastructure remain important hurdles. Second, while the article addresses patient-related problems such alarm fatigue and worry, a more in-depth examination of psychological implications, adherence and quality-of-life outcomes will reinforce its patient-centred viewpoint, which is supported by previous research on CGM use.^3^ Third, while the discussion on inpatient use of CGM emphasises limited evidence, it does not go into enough detail about emerging clinical trials that could soon affect practice guidelines. Furthermore, concerns such as interoperability, data integration into electronic health records, and digital health ecosystems are briefly highlighted and require further discussion.

To make this review more useful, future research might include cost-effectiveness assessments and implementation strategies from a variety of healthcare settings. Expanding organised training modules for general practitioners and multidisciplinary teams might also be beneficial. Furthermore, the addition of established clinical algorithms or decision-support systems may enable non-specialists in safely managing these technologies. Finally, a greater emphasis on long-term real-world results and patient-reported experiences might lead to a more complete knowledge of diabetes technology’s benefits and limitations.

Finally, this article makes an important contribution to the current diabetes care literature, notably in bridging the knowledge gap for general physicians. Addressing the aforementioned constraints and adopting a more global, patient-centred viewpoint would increase its effect and application.

## CRediT authorship contribution statement

**Prajakta Udmale:** Conceptualization, Writing – original draft. **Aparna Dixit:** Supervision. **Vaishali Khobragade:** Data curation, Formal analysis, Writing – review & editing.

## Funding

No funding was received for the preparation of this letter to the Editor. The authors did not receive any financial support from public, commercial or not-for-profit funding agencies for the research, authorship, or publication of this work.

## Declaration of Competing Interest

The authors declare that they have no known competing financial interests or personal relationships that could have appeared to influence the work reported in this letter. The authors have no financial or non-financial conflicts of interest to disclose.
